# Patellofemoral Pain Syndrome Risk Associated with Squats: A Systematic Review

**DOI:** 10.3390/ijerph19159241

**Published:** 2022-07-28

**Authors:** Pablo Monteiro Pereira, João Santos Baptista, Filipe Conceição, Joana Duarte, João Ferraz, José Torres Costa

**Affiliations:** 1 Associated Laboratory for Energy, Transports and Aeronautics (PROA/LAETA), Faculty of Engineering, University of Porto, 4200-465 Porto, Portugal; prof.monpe@outlook.com (P.M.P.); jasduarte@fe.up.pt (J.D.); ferraz.jhm@gmail.com (J.F.); 2 Center for Research, Education, Innovation and Intervention in Sport (CIFI2D/LABIOMEP), Faculty of Sports, University of Porto, 4200-450 Porto, Portugal; filipe@fade.up.pt; 3 Associated Laboratory for Energy, Transports and Aeronautics (PROA/LAETA), Faculty of Medicine, University of Porto, 4200-319 Porto, Portugal; zecatoco@sapo.pt

**Keywords:** PFPS, patellofemoral, anterior knee pain, chondromalacia, musculoskeletal disorder, prevention

## Abstract

Patellofemoral pain syndrome (PFPS) is highly prevalent; it can cause severe pain and evolve into progressive functional loss, leading to difficulties performing daily tasks such as climbing and descending stairs and squatting. This systematic review aimed to find evidence, in the literature, of squat movements that can cause or worsen PFPS. This work was based on the Preferred Reporting Items for Systematic Reviews and Meta-Analyses (PRISMA) statement, and its protocol was registered in PROSPERO (CRD42019128711). From the 6570 collected records, 37 were included. From these 37 articles, 27 present a causal relationship between knee flexion and PFPS, 8 describe a relationship, considering the greater existence of muscle contractions, and one article did not describe this relationship in its results. The main limitations stem from the fact that different studies used different evaluation parameters to compare the force exerted on the patellofemoral joint. Furthermore, most studies are focused on sports populations. After analysing the included works, it was concluded that all squat exercises can cause tension overload in the knee, especially with a knee flexion between 60° and 90° degrees. The main causal/worsening factors of PFPS symptoms are the knee translocation forward the toes (on the same body side) when flexing the knee, and the muscle imbalance between the thigh muscles.

## 1. Introduction

Patellofemoral pain syndrome (PFPS) is a widespread disease with a significant impact on society, with a 22.7% prevalence in the general population [1]. However, the values found by different authors differ substantially. It is possible to observe different prevalence rates in studies with specific populations. There were found prevalence values of 35.7% in professional cyclists during the competition season [2], 34.9% in workers at a large Iranian manufacturing company [3], 13.5% in the military [4], and 30% in drivers of selective waste collection [5].

PFPS is characterised by pain in the anterior region of the knee (peripatellar or retropatellar; stabbing, without irradiation, sometimes intermittent), worsening in squatting movements, climbing and descending stairs, and after long sitting periods [6,7]. It can be classified as MSD-2 or MSD-3 [8]. Several pathologies act as PFPS, such as anterior knee diseases (chondromalacia patellar, anterior knee pain syndrome, runner’s knee and patellofemoral tendon disease), whether or not they are associated with other pathologies such as knee osteoarthritis [6,9,10].

PFPS is a disease that can affect different population groups and occurs due to multiple factors [10,11,12], such as:

- Movement-related factors: Tasks performed with knee flexion above 60° and knee movement ahead of toes [10,13,14].
- Individual factors: Bone misalignments with little evidence [7,15,16] and muscle imbalances, increased strength of the hamstrings relative to the quadriceps, as well as anserine paw tendinopathy [15,17,18], as weakness in the quadriceps [19,20] and increased strength in the hip abductor muscles compared to other thigh muscles [12].
- Psychosocial factors: Anxiety and depression may be indirect risk factors for PFPS.

However, more research should be developed to prove these results [21].

Few studies have been developed in the occupational context. Consequently, there is little information available on workers’ exposure to the risk of developing PFPS as a consequence or aggravated by work activities. However, it is a fact that many occupational activities require squatting or similar movements in usual daily activities. Clinical approaches such as those used in sports medicine are often used to deal with the consequences of this problem.

The risk factors are related to changes in the movement kinetics of the tibiofemoral joint, caused by changes in the force exerted on the knee and hip joints. The risk of injury occurs when there is muscle imbalance of the trunk and hip or alteration of the kinetic movement of the feet and ankles [16,22,23,24,25,26]

Studies in rehabilitation have shown that bodybuilding physical activity, with protocols of exercises to strengthen the hips and quadriceps muscles, together with work tasks, are protective factors in decreasing PFPS incidence [17,19,27,28]. In the sports area, it is possible to find some studies with which it is possible to establish a direct relationship between the act of squatting and the occurrence of PFPS [28,29,30]. In other working environments, tasks involving loads movement with squat overuse can be observed, leading to PFPS.

With this background, this systematic review aimed to find in the literature and systematise the evidence that could relate the squat exercises to the emergence of PFPS.

## 2. Materials and Methods

This systematic review work followed the Preferred Reporting Items for Systematic Reviews and Meta-Analyses (PRISMA) Statement methodology [29]. The eligibility criteria, information sources, search, study selection, data collection process, data items, risk of bias in individual studies, summary measures, and synthesis of results required by the PRISMA 2020 Checklist [29] can be found in the Systematic Review Protocol [30] published in open-access. The protocol is registered in PROSPERO, under the code CRD42019128711 (www.crd.york.ac.uk/prospero/ (accessed on 15 October 2019)).

### 2.1. Eligibility Criteria

#### Search Characteristics

The search for information was carried out in three stages. In the first stage, a search of the available literature between 2014 and 2021 was carried out with a set of pre-defined keywords. Only articles, case reports, cohort and cross-sectional studies published in indexed journals, written in English, and published in peer-reviewed journals were considered.

The articles that met the eligibility criteria were selected in the second stage. In the third step, articles and other works published before 2014 were considered in the review through the snowball procedure [31]. In this procedure, a search was carried out for additional articles that meet the objectives through the references of all selected articles, authors, and respective research centres. In the third step, in the cases where there was no evidence of a peer review process (potentially weakening the quality and impartiality of the work), the authors assumed the role of reviewers.

### 2.2. Characteristics of Accepted Studies

#### 2.2.1. Participants

The search focused on studies developed on humans, healthy or with knee injuries, without gender or age restrictions. Studies developed in humans with prostheses orthoses and studies focused on animals or corpses were excluded.

#### 2.2.2. Type of Interventions

The interventions accepted and evaluated were those that addressed:

(a) Assessment of thigh, leg, and gluteal muscles through electromyography (EMG) and/or video analysis and/or motion kinetics;
(b) Evaluation of knee or hip joints during squatting movements by video and/or kinetic analysis;
(c) The patellofemoral pain syndrome and its relationship with the different movements of squatting or knees bending when evaluated by the methods described in (a) and (b);
(d) Pain assessment in patients with PFPS with the methods defined in (a) and (b);

Studies focused on other muscles or joints were excluded.

#### 2.2.3. Design of Accepted Studies

Studies within the following typologies and criteria were accepted: observational cohort and cross-sectional studies, case-control studies, before-after (pre-post) studies with no control group, case series studies, and controlled intervention studies, which evaluated the muscles responsible for movements related to the knee joint in any exercise involving squatting or flexion.

Studies that showed an increase in tensional overload in the patellofemoral joint were accepted, e.g., hyperactivity of the thigh muscles directly through EMG or indirectly through hyperactivity of the stabilising muscles.

### 2.3. Information Sources

The electronic databases searched were: Academic Search Complete, Scopus, Science Direct, Web of Science, PubMed, and Informaworld by Taylor & Francis and Medline (via PMC). In each database, the used filters were the year of publication (≥2014), type of document (articles and articles in press), type of source (journals and scientific publications), and language (English). The research was carried out from February to April 2021.

### 2.4. Search Strategy

The search strategy includes the combination of the following keywords: squat, “squat technique”, “musculoskeletal disorder”, “musculoskeletal disease”, knee, “knee pain”, syndrome, “patellofemoral pain”, “functional task”, and worker.

As a result, ten combinations were used as valid:

1. squat AND (“musculoskeletal disorder” OR “musculoskeletal diseases”) AND worker
2. “squat technique”;
3. squat AND syndrome AND worker;
4. squat AND worker AND Knee AND NOT osteoarthritis;
5. squat AND knee AND syndrome AND NOT osteoarthritis AND NOT “low back”;
6. “knee pain” AND worker
7. (“musculoskeletal disorder” OR “musculoskeletal disease”) AND “patellofemoral pain” AND NOT Osteoarthritis AND NOT “low back” AND NOT ankle;
8. squat AND “functional task”;
9. “functional task” AND “patellofemoral pain” AND NOT Osteoarthritis AND NOT ankle AND NOT “low back”;
10. “patellofemoral pain” AND worker AND NOT Osteoarthritis AND NOT ankle AND NOT “low back”.

### 2.5. Study Records

#### 2.5.1. Data Management

Data management occurred through the collection directly from virtual libraries. The selected studies from these sources were saved directly with the help of a reference manager software (Mendeley Reference Manager—Mendeley, London, UK).

After identifying the records, data extraction was performed one by one on a customised Excel table. In this table, every row corresponds to a different record and each column to one of the parameters extracted from each article.

#### 2.5.2. Selection Process

During the first screening phase, records obtained with each combination were automatically selected according to the year of publication (≥2014), document type (articles and forthcoming articles), source type (peer-reviewed journals), and language (written in English). After this first automatic screening, one of the researchers verified the works found with the alignment of the research and the review’s objectives based only on the screening of the title and abstract. If there were doubts regarding the interest of the record for the review after this preliminary verification, it would move on to the next selection phase.

After this first screening stage, the procedures were evaluated according to the eligibility criteria, requiring a full reading of each record.

Thus, the accepted studies were those that met all these minimum criteria:

- Inclusion of healthy humans or those with knee injuries but without prostheses or orthotics.
- Medical evaluation or by another qualified professional.
- Description of the relationship between knee flexion exercises such as squat and PFPS.
- Approach to the musculature of the thigh, leg, and gluteus, evaluated by EMG and/or video analysis and movement kinetics.
- Assessment of knee or hip joints during movement using video analysis and motion kinetics.
- Study of patellofemoral disease and its relationship with any type of physical exercise.

### 2.6. Data Collection Process

Each collected study was thoroughly evaluated qualitatively and quantitatively, analysing its results for the review. 

During the data collection process, the results of each study, presented in the form of an extensive description or a table, were considered, as well as its conclusions. All contents that could be interpreted with a causal link to the objective of the systematic review were considered.

The information extracted and included in a table were:

- General information: authors and year of publication.
- Population: athletes/workers and type of activities performed.
- Sample: size, sex distribution, mean age, and BMI.
- Study characteristics: objectives, evaluated parameters, procedures/methods, equipment and software, and conclusions.
- Parameters: type of exercise, muscles and joints involved, tensions in muscle tendons, applied interventions, results, and study characteristics.
- Quality assessment: possible risks of bias (selection, precision, information, researcher), reports (assessment of the overall quality of the study), external validity (assessment of whether the results of the study are generalisable), internal validity (assessment of bias due to study sample selection and/or confounding), and power (assessment of whether study results can be obtained by chance).
- Studies’ results and the direct or indirect relationship with the purpose of the review.

### 2.7. Prioritisation and Outcomes

In prioritising the articles and their results, greater relevance was given to the following three aspects:

Quantitative:

1. Corresponding to the direct analysis of tensional overload in the patellofemoral joint;
2. Tensional overload in the patellofemoral joint through maximum isovolumetric contraction via electroneuromyography.

Qualitative:

1. Pain reduction and mobility improvement in PFPS patients, after exercise intervention.

### 2.8. The Risk of Bias and Quality Assessment

The risk of bias (Table 1) was assessed independently by two reviewers, and, in case of doubt, a third reviewer intervened. The quality of selected articles was assessed using the National Heart, Lung, and Blood Institute (NHLBI) assessment tool [32].

Following the NHLBI, the evaluation criteria pursue issues related to the design of selected works. Methodological issues were addressed following five major groups. Each group presents a specific number of questions, having as a final result of the evaluation, Good, Fair, or Poor, for each of the evaluated studies: observational cohort studies, cross-sectional studies, and controlled intervention studies with 14 questions; case-control studies, before-after (pre-post) studies without control group with 12 questions; and case series studies with 9 questions.

Although there are no pre-determined criteria NHLBI, it was decided to consider the number of positive answers to the questions of each case, with Yes, >70% = GOOD, ≥50 and <70 = FAIR, and below 50% = Poor. All “Poor” works were excluded, as well as defined as works with a high risk of bias.

## 3. Results

**Figure 1:**
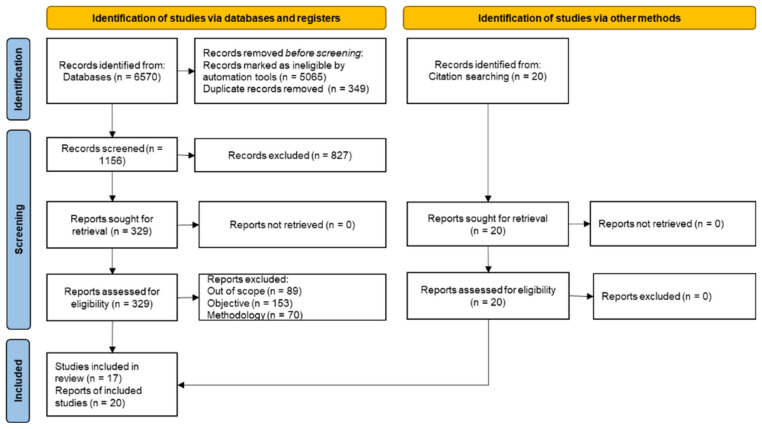
PRISMA Flow diagram of the research [29].

**Figure 2:**
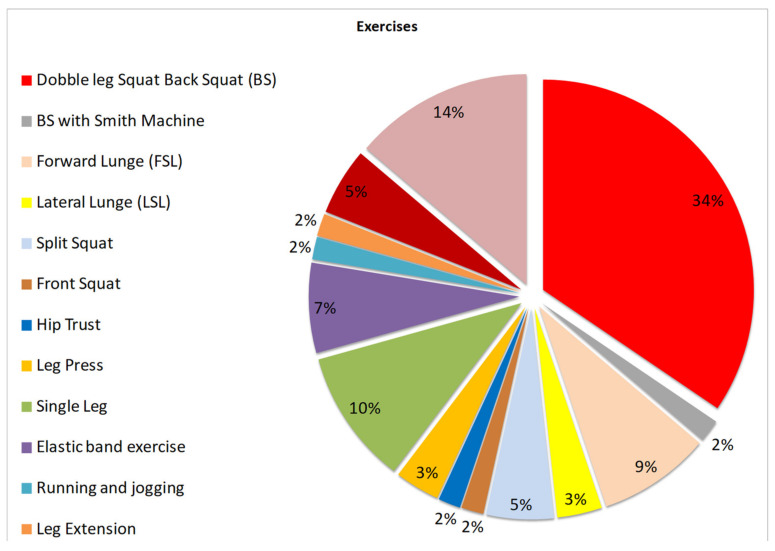
Types of exercise studied in the selected works.

The selection process results are shown in Figure 1 and Table A1, which present a compilation of the data extracted from selected studies. Figure 2 shows the different types of exercises described in the selected literature, and Table 1 summarises the risk of bias within the studies. Table 2 presents the results and their relationship with the review’s objective.

### 3.1. Article Selection

Per the PRISMA guidelines [29], 6570 items were initially found in the database searches. Then, the search engine filters, date, article type, font type, and language restrictions were applied, and 5065 articles were excluded. Additionally, 349 duplicate records were also removed.

In the screening phase, from the remaining 1156 articles, 827 were also excluded for being out of topic, with 329 remaining for retrieval. During this last step, 90 articles were excluded because the subject was not related to the scope of the review, 153 did not correspond to this review’s objectives, and 70 for methodological reasons.

Thus, it was possible to identify 17 relevant publications. Another 20 studies were obtained from cross-references through the snowballing methodology [31], mainly published before 2014. Figure 1 provides an overview of the number of articles identified in each step delimited by the PRISMA methodology. The results were obtained unanimously among the evaluators.

### 3.2. Risk of Bias

The quality of a review work depends on the quality of the studies on which it is based. To verify the quality of the studies selected through the selected NHLDI evaluation tool, there were 28 observational cohort and cross-sectional studies, three case-control studies, one before-after (Ppre-Ppost) study with no control group, one case series study, and four controlled intervention studies. As a final analysis result, 35 were classified as good articles and two as fair works (see Table 2 and Table A2 for details).

Other criteria, such as stabilising muscles in squatting movements, are accepted in the literature. However, they present indirect characteristics when trying to obtain a direct answer to the review question. Moreover, different types of exercises (interventions) applied in the studies were explicitly evaluated for the knee joint.

In Table 2, the presence of a causal relationship (green cells) was considered whenever the article presented evidence of tension overload on the patellofemoral joint or knee joint by one of two means: (1) joint tension overload (direct) or (2) muscle tension overload (indirect), despite reporting or not of PFPS symptomatology.

In the article, an unclear causal relationship (yellow cells) was considered whenever the peaks of force (stress) on the joint directly or indirectly via musculature were assessed, but no causal nexus relations were evaluated. In this case, the link was considered unclear through the author’s discussion, as well as scientific evidence.

Moreover, it was considered that there was no causal link (red cells in Table 2) when there was no relation between the studied movement as aggravating or generating PFPS in the article.

The results show that from the 37 studies accepted in the review, 27 showed a causal link, 9 showed an unclear relationship, and one did not find any causal link.

## 4. Discussion, Summary of Evidence

The research findings demonstrated a significant variability of squat exercises between the selected studies. However, 34% of the works used the bipedal squat (Figure 2).

In general, the use of sports articles occurred due to the scarcity in the scientific literature of studies that evaluate the biomechanical factors related to PFPS in the work context. Thus, associating knee flexion with patellofemoral joint overload would only be possible through sports-based studies.

Thereby considering the different risk factors for the emergence or worsening of PFPS [10], the knowledge about the tension overload on the patellofemoral joint force (PFJF) is discussed in three main aspects in this review.

The first one is based on the knowledge about the degrees of knee flexion, considering its anterior translational movement over the anterior tibial line as the main factor of PFPS worsening [22,40,45,49,53,54,65].

Second, as scientific knowledge on the subject has increased, the perception of PFJF has been quantified through EMG. Thus, the studies evaluated muscle activation patterns, followed by understanding the muscles that could potentially protect the joint from the movement of flexing the knee [7,15,18].

Finally, as a third point, considering the posed research question, the tension overloads on the knees are compared following the evaluation models applied in different types of squats. In these cases, it is observed that there is an overload of the patellofemoral ligament in diverse degrees of knee flexion, mainly between 60° and 90° degrees [22,24,33,49,53,57,62].

Some studies have attempted to identify overload patterns when there was a change in the distance between the feet, in the frontal plane, and during the squatting movement, but no difference in the patellofemoral ligament overload has been demonstrated with the distance between feet [22,35,50]. However, other authors describe that there would be tension overload on the patellofemoral joint when there is a contraction of the quadriceps muscle, regardless of different distances [22,25,26,36].

After analysing the different authors, the two main aggravating factors of PFPS tension overload on the patellofemoral ligament are:

- The anterior translation of the knee with the anterior tibial line in front of the ipsilateral toes line during any squat type [22,24,33,46,49,53,65]. Thus, a difference of strength on the patellar tendon of 18.8% compared to 11.5% was found during the forward lunge squat exercise when the anterior tibial line was translocated from the knee to the front tibial line anterior to the ipsilateral toes line [34].
- Muscle imbalance of the thigh muscles, posteroanterior, and stabilisers such as gluteus medius and vastus medialis oblique muscle (VMO) demonstrated via EMG by the ability to contract. According to some studies, this imbalance was the cause of the most significant pain in patients with PFPS compared to people without the syndrome. It is, therefore, considered one of the main aggravating factors [37,38,60].

It is considered that strengthening the VMO and middle gluteus muscles is a protective factor concerning PFPS [43]. Thus, comparing the muscle activation patterns in individuals with and without PFPS, it is verified the existence, in the first ones, of an imbalance in the muscular activation patterns of the single-leg movement [48].

In subjects with PFPS, the muscles activated sequentially during single-leg stance are gluteus medius, gluteus maximum, vastus lateral muscle (VL), and then VMO. On the contrary, healthy subjects activate gluteus medius, VMO, VL, and gluteus maximus. Therefore, comparing the choice between closed kinetic chain (CKC) with open kinetic chain (OKC) exercises to improve strength, it can be observed that the first ones present a more significant strength gain and increased thigh musculature [41]. At the same time, CKC exercises also improve kinematics and pain symptoms in the population with PFPS who perform such exercises compared to sedentary ones [42,55,63,66].

There was no statistical difference in tension on the knee joint in free-weight squat (double squat) and split squat exercises. Thus, it was considered that the anterior translation of the tibia and the muscle imbalance in both movements were the main factors of worsening pain and knee joint overload [33].

When the forward lunge (FL) and lateral lunge (LL) were compared, it was possible to identify that the biomechanics of the movements were different, with the FL being more related to the knee overload movement and the LL to the ankle movement [23,24].

When comparing the walking lunge with the split squat, both using overload weight, it was found that there was a greater need for muscle activation to stabilise the joint during the walking lunge exercise with a contralateral dumbbell, followed by the split squat activity with an ipsilateral dumbbell. It is possible to identify that the torque of the knee was bigger in the walking lunge than in the split squat [39].

A single study compared the back squat and the front squat. It was demonstrated that the front squat presents muscle activity when squatting with 1RM, similar to the back squat, and needs less load to generate similar responses. It also offers less risk of injury when protecting the intervertebral spaces and the patellofemoral and tibiofemoral joints [47].

Studies comparing muscle strength using the free weight squat and Smith machine squat methods demonstrated that free weight squat presents better strength gain in the thigh muscles [52]. However, increased musculature increases PFJF overload [25] due to instability during movement [52].

When comparing leg extension exercise with free weight squat, the same VMO/VL ratio was found. However, in the MVIC (maximum voluntary isometric contraction) of the VMO, it was observed that the maximum quadriceps forces in squats were higher than the leg extension exercise [38].

However, in the same study, it has also been shown that there is a possibility for worsening patellofemoral pain. It can occur in the contraction of hip abductor muscles during squat exercise with the external hip rotation. As a consequence of these movements, the isolated response of the VL and the middle gluteus can grow, generating an excessive muscular increase of the gluteus medius and the VL. Consequently, this creates greater lateral traction force on the patella increasing pain [38].

The muscle activation pattern in the back squat (with weight) and barbell hip thrust showed that the hip thrust muscle activity was superior to the back squat. 

Nevertheless, with minimal knee joint overload, this exercise can be a possibility of application in the context of rehabilitation [25,51,58]

For a practical approach to injury prevention and reducing pain symptoms, the importance of muscle strength in the lower limbs is the main preventive factor, as well as muscle balance [9,15,17,18,23,59,60,62,64]. With this knowledge from rehabilitation, the barbell hip thrust is safer for the kinetic movement, being an alternative in the treatment [25,37,64].

Despite not having statistical differences, some exercises such as single-leg, double leg, or even hip abductors strength with or without load training seem to reduce the risk factors for the onset of PFPS [44,61].

The results shown in Table 2 allowed better visualisation of the causal link between the squat exercise related to knee flexion and PFPJF. Despite the great variability of the squats studied, it was possible through each article’s direct and indirect results to identify the relationship between joint overload and knee flexing.

Considering the evidence presented on the analysed exercises, the importance of using the squat in the first moment of rehabilitation of patients with PFPS can be questioned. Similarly, the task with excessive squatting movements in the work context can be considered a risk factor for PFPS worsening [22,56,58,61].

## 5. Conclusions

The analysis of the selected studies suggests that all squat exercises generate overload on the patellofemoral joint, which may cause the occurrence or worsen of PFPS (Table A1). This syndrome may or may not be symptomatic. It occurs mainly due to tension overload on the patellofemoral ligament in knee flexion between 60° and 90°, and when, during knee flexion, the vertical line passing through the anterior region of the tibia crosses the vertical line passing through the tips of the toes (on the same side) [22,40,45,49,53,54,65].

The imbalances between the posterior and anterior thigh muscles are directly related to the symptomatology. However, it was impossible to compare all types of squats due to the different parameters used by the different researchers.

It was possible to verify that squat movements in which the knee passes in front of the ipsilateral line of the toes are the main aggravating factor of tension overload on the PFJ, having a direct relationship with PFPS.

Finally, considering the exercises with greater instability, comparing the bipedal squat and the walk lunge (forward lunge), the bipedal squat is safer for the movement due to the balance in its execution. However, they do not present significant differences regarding the tension overload in the PFJF. The same goes for the Smith machine double squat and the barbell squat, making the Smith machine exercise the safest.

According to Contreras et al. (2015) [26], the possibility of using exercises other than the squat (hip trust exercise, for example) for muscle strengthening is a form of joint protection, with results similar to the strength gain of the squat exercise.

## 6. Significance for Practice

All types of squats can be harmful to people with PFPS. Nevertheless, in the context of rehabilitation, intervention in individual risk factors can reduce pain in daily tasks and increase functional capacity.

Thus, the PFPS prevention modes identified in the present study were the correct kinematic, dynamic valgus, internal rotation of the femur during squat activities, overuse without concomitant muscle activity, and muscle imbalances in the lower limbs.

Maintaining the individual doing weight training using protocols for strength training of the hip and quadriceps muscles, along with the biomechanics of movement, are the main protective factors of the syndrome.

## 7. Limitation

The authors acknowledge that the evaluation parameters were different in the different studies. The evaluation of tension overload in the patellofemoral joint in the different squat exercises, performed with other methods, made it difficult and limited the analysis and comparison of results. Another limitation is the need for an indirect analysis of patellofemoral joint overload and few studies related to PFPS problems in occupational environments.

A limitation from a methodological point of view is the existence of studies with a small sample and few prospective studies focused on PFPS.

## Figures and Tables

**Table 1:** Summary of Overall Risk of bias within studies and quality assessment.

References	Quality Tool Assessment NHLDI *	Study Authors	Quality Tool Assessment NHLDI *	Study Authors	Quality Tool Assessment NHLDI *
[22]	G	[23]	G	[24]	G
[25]	G	[26]	G	[33]	G
[34]	G	[35]	G	[36]	G
[37]	G	[38]	G	[39]	G
[40]	G	[41]	F	[42]	G
[43]	G	[44]	G	[45]	G
[46]	G	[47]	G	[48]	G
[49]	G	[50]	G	[51]	G
[52]	G	[53]	G	[54]	G
[55]	G	[56]	G	[57]	G
[58]	G	[59]	F	[60]	G
[61]	G	[62]	G	[63]	G
[64]	G				

Legend: * NHLDI—National Heart, Lung, and Blood Institute: G—Good, F—Fair.

**Table 2:** Relationship between selected studies and the review objective.

Refs.		[22]	[23]	[24]	[25]	[26]	[33]	[34]	[35]	[36]	[37]	[38]	[39]	[40]	[41]	[42]	[43]	[44]	[45]	[46]	[47]	[48]	[49]	[50]	[51]	[52]	[53]	[54]	[55]	[56]	[57]	[58]	[59]	[60]	[61]	[62]	[63]	[64]
(a)	Y																																					
	N																																					
	U																																					
(b)	Y																																					
	N																																					
(c)	Y																																					
	N																																					

(a) Does the article show any evidence of the causal link? (b) Are there Knee and Patellofemoral Joint Stressed by over tension? (c) Did the results evaluate the symptoms of PFPS? Y—Yes; N—No; U—Unclear.

## Data Availability

Not applicable.

## Appendix A

**Table A1:** Studies characteristics.

Ref.	Study Characteristics		Population						Study Result							
	Type of Exercise	Assessed Parameters	Athletes/Workers	Characteristics	Gender		Mean Age (Years)	BMI Kg/m^2^	Relationship with the Objective of SR							
					Sample	Ctr			Direct							Indirect
[22]	1—Back Squat (Double squat-SQ), Wise squat (WS), narrow squat (NS) 2—Leg Press (LP), Foot different position (high (LPH), Lower (LPL), Wise (WS), Narrow (NS).	Kinetics/Kinematics/Force Muscle/Force on Join/Force Tendon (Anterior cruciate ligament tension: ACL, posterior cruciate ligament: LCL, Tibiofemural tension: TB, Patellofemoral tension: PF)	Physically fit and Healthy subject	Lifters experienced in performing the squat and Legg Press	Male: 10	NA	Male: 29.6 ± 6.5	Male: 29.84	Patellofemoral joint stress at knee flexion— Megapascal Pressure Unit (MPa) 20°—0.92 MPa, 30°—1.98 MPa, 60°—7.00 MPa, 90°—10.21 MPa							NA
[23]	Forward Lunge (FL) Lateral Lunge (LL)	“muscle activation patterns joint moments, powers, impulse mechanical energy expenditure.”	Healthy Subject	NM	Male: 9 Female: 11	NA	Overall: 75 ± 4.4	Overall: 28.49	Knee Peak Power (W·kg^−1^)							Peak knee power generated during FL was 46.2% greater than during the LL
[24]	Forward lunge (FL) (with and without a stride) Side (Lateral) lunges (LL) (with and without a stride)	Muscle activation patterns Kinematic Force	Healthy Subject	NM	Male: 9 Female: 9	NA	Male: 29 ± 7 Female: 25 ± 2	Male: 25.77 Female: 22.22	Patellofemoral force (N) values approximate/by knee angle for knee flexing phase							NA
									angle	Without stride			With stride			
										FL	LL		FL	LL		
									0° 10° 20° 30° 40° 50° 60° 70° 80° 90°	69 159 207 356 628 1059 1524 1944 2161 2185	46 127 194 332 573 926 1533 2009 2493 2668		74 220 301 459 732 1150 1683 2058 2344 2411	49 80 120 242 481 856 1378 1896 2267 2419		
[25]	Back Squat (BS) Barbell Hip Thrust	Range of Motion (ROM) to Knee and Hip Joint. Kinematic analyses movement Muscle activation patterns Upper Gluteus Maximus-(UGM) Lower Gluteus—(LG) Biceps Femoris—(BF) Vastus Lateralis—(VL)	Healthy subject	Subject with strength training experience	Female: 13	NA	Overall: 28.9 ± 5.11	Overall: 21.97	Comparison between EMG-verified Thigh Muscles Peak Strength, Back Squat and Barbell Hip Thrust							NA
									Back squat	UGM	LG		BF	VL		
										84.85 ± 42.91	129.60 ± 60.45		37.50 ± 18.39	243.92 ± 121.63		
									Hip Thrust	171.75 ± 90.99	215.85 ± 83.76		86.87 ± 38.81	215.83 ± 193.89		
[26]	Back Squat with load and without load	- patellofemoral joint reaction forces - patellofemoral joint stress.	Healthy Subject	NM	Male: 6 Female: 9	NA	Overall: 26 ± 5	Overall: 24.69	Patellofemoral joint stress at knee flexion—90°							“The results indicate that patellofemoral joint stress increases linearly with increasing knee flexion angle and decreases with decreasing knee flexion angle.”
									Concentric phase				Eccentric phase			
									load	unload			Load	unload		
									13.0 MPa	9.3 MPa			13.06 MPa	9.06 MPa		
									Descending				Ascending			
									FL	LL			FL	LL		
									0.71 (0.40)	0.52 (0.26)			0.76 (0.35)	0.48 (0.25)		
[33]	”Split squats with an additional 25% body weight load applied using a barbell.”	Range of Motion (ROM) to Knee and Hip Joint. Kinematic analyses movement. Kinetic movement to split squat	Healthy subject	Students	Male: 5 Female: 6	NA	Overall: 24.9 ± 2.5	NI	Front leg relation between tibial angle and knee tension force (N∙m/kg, positive for external flexion)							NA
									leg length	60°	75°		90°	105°		
									L-1	1.46 ± 0.14	1.38 ± 0.17		1.19 ± 0.22	NM		
									L-2	1.40 ±.17	1.33 ± 0.20		1.14 ± 0.19	NM		
									L-3	1.28 ± 0.12	1.19 ± 0.17		1.13 ± 0.21	0.80 ± 0.16		
									Split squat step lengths of l1 = 55% of leg length (ll), l2 = 70% ll, and l3 = 85% ll and four tibia angles (a) were evaluated							
[34]	“Forward Lunge—with the Knee translated in front of the toes (FSL-FT) Forward Lunge—with the Knee translated behind the toes (FSL-BT).”	- Peak force by Knee kinematics Kinect - muscle activation patterns (EMG)	healthy subject	Volunteers	Female: 25	NA	Overall: 22.69 ± 0.74	Overall: 21.55	The peak patellar tendon stress, stress impulse, quadriceps force, knee moment, knee flexion, and ankle dorsiflexion angle were significantly greater during the FSL-FT than the FSL-BT. The peak patellar tendon stress rate did not differ between the FSL-FT and FSL-BT							NA
[35]	Back Squat with weight	Anthropometric data./3D knee kinematics. (Tibiofemoral joint kinematics.)/“Ground reaction forces (A—Tibiofemoral joint moment, B—Patellofemoral joint reaction force) Patellofemoral joint stress.”	Healthy Subject	Collegiate women athletes	Female: 5	NA	Female: 19 ± 1.4	Female: 23.49	Patellofemoral joint stress at knee Flexion: 72.8° = 4319.2 N 91.5° = 5065.7 N 109.7° = 5097.1 N							NA
[36]	Squatting Running	1—joint kinematics 2—kinetics 3—muscle forces pPFJS indicates the peak patellofemoral joint stress (MPa); PFJS-TI = patellofemoral joint stress time integral (MPa s); (pQF) indicates the peak quadriceps force (pQF)	Healthy subject	Subject with strength training experience	Female: 11	NA	Overall: 22 ± 1.8	Overall: 22.47	Squat trials	Methods						NA
										ID—Inverse dynamics			IDO—Inverse dynamics and static optimisation			
									PPFJS	9.81 (sd 3.36)			17.06 (sd 4.34)			
									PFJS-TI	7.51 (sd 1.98)			12.87 (sd 2.33)			
									pQF	3.81 (sd 0.72)			5.16 (sd 0.82)			
[37]	“Squat with three displacements. Displacing the Knee anteriorly (AP malaligned), displacing the Knee medially (ML malaligned). Squats with control alignment (control).”	1—electromyographic muscle activation during a neutrally aligned squat	healthy subject	Volunteers	Male: 9 Female: 19	NA	Overall: 21.5 ± 3	Overall: 22.73	The misaligned movement, such as the anterior translation of the tibia concerning the foot on the same side, has been shown to be the moment of greatest knee joint overload, requiring excessive activity of the knee's stabilising muscles. It has been shown that the moment of decreased muscle activity in the quadriceps is the moment when the contact forces between the patella are greatest.							NA
[38]	“Exercise 1–3 with an elastic band, hip abductor exercise, hip external rotator exercise. Leg extension, Two-legged Squat (Double Legged).”	1—muscle activation patterns (EMG). (VL, VMO, and gluteus medius muscles)	Subject with PFPS	Volunteers Students	Female: 30	NA	Overall: 24.93 ± 4.91	Overall: 22.95	NA							VMO muscle activation was induced during the closed kinetic chain knee movements, and the VL/VMO was 1 in Close and Open chain exercise
[39]	(WL)Forward (Walking) lunge (A)Dumbbell = Ipsi-lateral (B)Dumbbell = Contra-lateral (SS)Split Squat	“Range of Motion (ROM) to Knee and Hip Joint, Kinematic analyses movement, Kinetic by movement to split squat, Muscle activation patterns, PT = peak torque obtained during a 5-second isometric contraction at the stated joint angle” VM—Vastos Medialis Muscle, VL—Vastos Lateralis Muscle	Athletes and Healthy subject	Athletes: Powerlifters competing in the Czech championships during	14	14	InT: 30.02 ± 5.60 C: 28.35 ± 5.71	InT: 27.22 C: 24.01	NA							This finding suggests that contralateral WLs may increase the muscle imbalance between the VM and VL, especially in RT individuals. Alternatively, contra-lateral WLs appear to benefit Gmed and VL strengthening if such strengthening is the aim of a training program.
[40]	“Back Squat with 3 different distances to foot and 9 different positions”: “Narrow (NW)—0°, 21°, 42° Hip distance (HS)—0°, 21°, 42° Wide (WS)—0°, 21°, 42°.”	1—Kinematics 2—range of motion and moments (9 different moments)	Physically fit subject	Volunteers do work out on gym (novice and experienced)	Novice: (Male: 10/Female: 11) Experienced: (Male: 10/Female: 11)	NA	Novice: 25 ± 6 Experienced: 25 ± 5	Novice: 22.41 Experienced: 22.76	NA							We would recommend that a moderate foot placement angle (approximately 20°) in combination with a moderate stance width (with feet approximately shoulder-width apart) should be used, and extreme positions would be avoided
[41]	Straight leg raise—freeload (OCK) semi-squat exercises—bodyweight (CKC)	- Q angle - Thigh circumference - Degree of anterior knee pain - crepation, - Isometric maximal voluntary contraction force (IMVC)	Students with PFPS	University students with a diagnosis of patellar chondromalacia	Female: 16 CKC Female: 16 OKC	NA	NI	NI	“The results indicate a significant increase of thigh circumference in the semi-squat group at 5 cm (*p* = 0.002) and 10 cm (*p* = 0.01) above the patella compared to the SLR group”.							“The exercise may improve patellofemoral joint performance by improving quadriceps muscle strength and correcting patellar alignment.”
[42]	1—Sling-Based Open Kinetic Knee Extension Exercise (1) 2—Sling-based closed kinetic knee extension exercise (2) 3—Sling-based hip adduction exercise (3)	Muscle activation patterns (MVC), maximal voluntary contraction	Subject with PFPS	Rehabilitation Center patients	Male: 30 Female: 30	NA	Male: 21.19 ± 0.68 Female: 21.12 ± 0.74	Male: 21.16 Female: 20.26	Electromyography analysis of MVC for open and closed kinetic knee extension and hip adduction exercises							For Chang, “the main factor causing patellofemoral pain is an imbalance of VMO and VL muscle, leading to excessive lateral tracking of the patella.”
									Muscle	Exercise						
										(1)			(2)	(3)		
									VMO	0.60 ± 0.20			0.71 ± 0.20	0.54 ± 0.13		
									VL	0.76 ± 0.12			0.72 ± 0.13	0.56 ± 0.14		
									VMO: VL	0.80 ± 0.31			1.00 ± 0.28	1.02 ± 0.35		
[43]	“(1) short arc quad with neutral hip position (SAQN), (2) short arc quad with externally rotated hip position (SAQER), (3) medial tibial rotation (MTR) (4) hip adduction (HA)”.	1—muscle activation patterns (EMG)	Worker with PFPS	Automotive workers	Male: 11	NA	Overall: 38.45 ± 12.73	Overall: 22.80 ± 4.69	NA							Mean iEMG activity of VMO muscle is more than that of VL muscle during all biomechanical rehabilitative exercises. However, one-way ANOVA with repeated measures indicates that mean iEMG activity of VMO muscle is significantly higher than that of VL muscle during SAQER only
[44]	“Single-Leg Squat, Single-Leg Landing, Hip abductor strength Free, Hip Abductors strength with load, This method sequentially challenges the control of the knee position during squatting by gradually progressing from double-leg to single-leg squatting”.	1—Kinematics to the joint Knee, hip.	Worker	Military volunteers	Male: 11 Female: 4	Male: 10 Female: 5	AG: 30.3 (8.8) Control: 29.6 (9.7)	AG:30.3 0 Control: 23.30	NA							NA
[45]	Squat: Two variation knees before the toes (SBT) knees to go past the toes (SPT).	patellofemoral joint force (PFJF) and quadriceps force (QF) patellofemoral joint reaction force (PFJRF) centre of pressure (COP) knee flexion range of motion (ROM) A/P—anteroposterior	healthy females	Volunteers	Female: 25	NA	Overall: 22.69 ± 0.74	Overall: 21.45	The PFJS, reaction force, and quadriceps force magnitudes were higher (*p* ˂ 0.001) during SPT than during the SBT technique.							NA
[46]	Forward lunge with a long step (FLF) Forward lunge with a short step(FLS)	Muscle activation patterns Kinematic Force	Healthy Subject	NM	Male: 9 Female: 9	NA	Male: 29 ± 7 Female: 25 ± 2	Male: 25.77 Female: 22.22	Patellofemoral force (N) values approximate/by Knee angle for knee flexing phase							NA
									angle	Without stride			With stride			
										FLF	FLS		FLF	FLS		
									0° 10° 20° 30° 40° 50° 60° 70° 80° 90°	69 159 207 356 628 1059 1524 1944 2161 2185	93 144 233 377 629 1051 1660 2335 2836 3039		106 212 306 440 688 1106 1585 2172 2567 2648	62 97 147 298 573 1006 1585 2172 2567 2648		
[47]	Back Squat Front Squat	muscle activation patterns (EMG) kinematics of Knee and hip joints (Video)	Athlete	NM	Male: 20	NA	NM	NM	EMG activities between back squats and front squats during the descending and ascending phases, performed with 1RM loads							“Instead of the load, the level of effort in volunteers’ muscle actions determine the degree of motor unit activity (Carpinelli, 2008); consequently, the level of EMG signalling increases. In addition, higher loads can increase the strain on the patellofemoral joints, increasing the risk of injury during the squat.”
									Muscle	descending phases			ascending phases			
										Back Squat	Front Squat		Back Squat	Front Squat		
									Rectus femoris	37.9 ± 12.1	46.4 ± 24.4		36.0 ± 13.8	46.7 ± 19.4		
									Vastus medialis	48.3 ± 14.3	53.1 ± 19.3		49.3 ± 13.9	58.9 ± 17.1		
									Vastus lateralis	45.9 ± 13.9	48.0 ± 15.8		48.5 ± 17.2	56.2 ± 22.2		
[48]	single-leg squat single-leg stance	1—muscle activation patterns (EMG)	Subject with/without PFPS	Volunteers	Male: 18	Male: 18	with PFP: 24.2 ± 4.4 controls: 213.5 ± 3.8	PFP: 23.8 Control: 23.9	Compared to healthy subjects, males with PFP demonstrated altered gluteus medius, VMO, and VL muscle activity during single-leg stance and single-leg squat.							NA
[49]		1—Muscle activation patterns 3—Kinematic	Healthy Subject	NM	Male: 9 Female: 9	NA	Male: 29 ± 7 Female: 25 ± 2	Male: 25.00 Female: 22.20	Patellofemoral joint stress (MPa) approximate							“The primary cause of the greater patellofemoral force and stress between 90- and 70-knee angles in the wall squat short compared with the one-leg squat was greater quadriceps force during the wall squat short.”
									angle	Descending			Ascending			
										SL	WSL	WSS	SL	WSL	WSS	
									60° 70° 80° 90°	5.3 6.6 7.0 7.2	4.5 6.7 7.5 7.3	4.5 6.6 7.6 8.0	5.8 7.3 7.0 7.5	8.0 9.5 8,3 7.5	6.3 8.8 9.5 9.2	
[50]	“Two-legged (double-legged), Squat (three squat depths (20°, 50° and 80° of knee flexion) while following three knee movement paths (neutral, varus or valgus).”	Kinematic for Squat with three variation muscle activation patterns (EMG)	Healthy subject	NM	Male: 7 Female: 11	NA	Overall: 22.39 ± 2.25	Overall: 22.49	Muscle activation, vastus medialis obliquus (VMO) and vastus lateralis (VL) and VMO: VL ratio (neutral)							NA
									Angle	VMO			VL	VMO: VL		
									20°	31.91			138.76	1.35		
									50°	74.45			267.52	1.69		
									80°	146.16			512.68	1.84		
[51]	Single-limb squat	Kinematic muscle activation patterns (EMG) Pain	Women not athletes with PFPS and Hip Joint Pain HIP (HJP)	Volunteers	−20 PFPS −14 HJP	−20 knee Control −13 Control Hip	18–40 years	17.82–32.30	Greater external rotation of the knee was correlated with greater knee pain in patellofemoral pain syndrome. All women with dynamic valgus movement had pain when performing single-leg squat exercises.							NA
[52]	DoubleLegged—two variation Free weight squat Smith machine squat.	1—Muscle activation patterns 3—Kinematic	Healthy Subject	Subject with strength training experience (2–5 years)	Male: 3 Female: 3	NA	Overall: 22 ± 1.2	Overall: 24.85	“The free weight squat elicited a 34% higher EMG MAV from the gastrocnemius, a 26% higher EMG MAV from the biceps femoris, and a 49% higher EMG MAV from the vastus medialis compared to the Smith machine squat.”							“Our finding of a higher biceps femoris and gastrocnemius activity during the free weight squat may be attributed to the increased role that the knee flexors play in stabilising and supporting the ankle, knee, and hip joints in a more unstable.”
[53]	Two-legged Squat (Double legged) 1—Control, two-legged squat 2—Valgus dynamic Squat 3—Knee Anterior Displacement Squat	Kinematic for Squat with three variation Torque to force by knee joint, hip joint, trunk joint, ankle joint	Healthy subject	Volunteers	Male: 11 Females: 19	NA	Overall: 21.4 ± 3	Overall: 22.77	NA							The overload in W/kg on the knee presents greater intensity in the knee flexion movement with anterior malaligned, followed by medial malaligned concerning the control (neutral).
[54]	single-leg squat (SLS) step-down task (SDT) PENS (electrical neuromuscular stimulation)	Kinematic muscle activation patterns (EMG) Pain	Women not athletes with PFPS	Volunteers	8	8	23.3 ± 4.9	24.1	Rehabilitation with neuromuscular activation via PENS (electrical neuromuscular stimulation) was beneficial in the patient with PFPS, decreasing the activation of the stabilising muscles in the single-leg squat movement and decreasing pain when performing the movement.							NA
[55]	“1—Semi Squat with hip adduction and internal rotation-CKC 2—Quadriceps isometric in supine position-OKC 3—Terminal knee extension with elastic band—KC 4—Terminal knee extension in supine position OKC 5—Adductor squeeze in crook lying (squeeze the ball) (odd weeks)—CKC 6—Hip adduction in lateral decubitus position (even weeks)–OKC”	1—Subjective symptoms, 2—functional performance, 3—knee flexion during stair ascent and descent by the patients. 4—VAS—Visual Analog Scale 5—Kujala Questionnaire	Patients	Patients with PFPS	Male: 5 Female: 9	Male: 5 Female: 9	Interv.: 25.7 ± 2.6 Control: 26.8 ± 2.3	Interv: 23.56 Control: 23.19	Clinical outcomes in the patients with patellofemoral pain syndrome (value).							NA
									Assessment		Group Exercise		Control			
									Intervencion		Pre	Pós	Pre	Pós		
									Pain while ascending (VAS)		49.8	19.6	51.3	54.4		
									Pain while descending (VAS)		51.3	19.8	50.2	54.2		
									AKPS (Kujala)		65.2	85.8	61.5	59.6		
[56]	“1—Squat (until 60° degree) 2—Intervention exercise: table hip abduction with 60°, 15° of hip extension and 30° of external hip rotation with the Knee extended. The subject returned his lower limb to a parallel position.”	Squat range of motion Maximal isometric voluntary contraction (MVIC)	Healthy subject	Volunteers	Female: 11	NA	Overall: 23.88 ± 2.64	Overall: 21.75	NA							During the squat exercise, the average activation ranged from 18.43% to 27.75% MVIC. The ability to activate the gluteus medius increased by 19.99 points after the adaptation exercises. However, the increase in isometric exercise varied with average activation ranging from 24.16% to 25.30% and from 18.43% to 19.70% in the dynamic exercise (squat). Thus, the muscular activity of isometric exercises is greater.
[57]	Knee Flexion 0°–30°	Noninvasive patellar tracking study using a “C”-arm computed tomography (CT) scanner	Women not athletes with PFPS	Volunteers	12 women 6 men	8 women 10 men	I: 31 ± 9 Control: 39 ± 15	NA	“Were observed for patellar proximal-distal shift (PTy) during NWB0°, patella flexion (PF) during WB30°, and anterior-posterior patella shift (PTz) during NWB0°, WB0°, and WB30° on the CT scan.”							NA
[58]	Single–leg squat (SLS) Split–Squat (SS) Squat (Double legged)	Kinematics to exercise movement in water (“included the average angular displacement and speed for each phase) The total range of motion and peak velocities.”	Healthy subject	University students	Male: 14 Female: 11	NA	Overall: 22.3 ± 2.9	Male: 24.03 Female: 22.01	NA							The study showed a lower intensity of exercises in the water and improved mobility. Improvement in movement technique was also demonstrated as a function of immersion concerning land exercise.
[59]	single-leg squat Back Squat Barbell Hip Thrust	Kinematic Pain	Men with PFPS	Volunteers	1	0	25	23.39	Maintaining muscle mass is undoubtedly the best path to healthy joints, preventing muscle injuries and increasing the ability to withstand muscle overuse and muscle overload in daily tasks. After one year of training with different physical exercises, the end of pain was evident.							NA
[60]	Squat Jump with Countermoviment with barbell weight −70% 1RM (A—jump squat) Squat Jump with Countermoviment with free loan, and free swing movement by arms (B vertical jump) Hang Lift (Olympic-style lifts—(C) power clean)	Kinetics Kinematics muscle activation patterns of the countermovement jump, the power clean, and the jump squat	Athletes	University student	Male: 10 Female: 10	NA	Male: 22.7 ± 3.7 Female: 20.4 ± 0.7	Male: 26.60 Female: 22.60	Force in Muscle Vastus Medialis (Quadriceps) by exercise. 1—power cleans (17,254 N s^−1^), 2—jump squat (7920 N s^−1^) 3—vertical jump (9465 N s^−1^)							NA
[61]	“1—isometric squat exercises (with) ball”	“muscle activation patterns (EMG) The muscle activity and the ratio of muscle activity were measured”.	Healthy Subject	Students	Male: 24	NA	Overall: 26.04 ± 2.19	Overall: 23.71	“An efficient squat exercise posture for preventing the patellofemoral pain syndrome is to increase the knee joint bending angle on a stable surface. However, it would be efficient for patients with difficulties bending the knee joint to keep a knee joint bending angle of 15 degrees or less on an unstable surface.”							NA
[62]	overhead squat	Electromyography and kinematic	healthy females	Volunteers	8 (MKD)	14	18–28	MDK 22.62 ± 1.85 Control: 21.82 ± 1.28	We observed medial knee displacement (MKD) that exhibit greater muscle activity in the following muscles: adductor magnus, biceps femoris, vastus lateralis, and vastus medialis muscles during the eccentric phase of the overhead squat. Regarding the kinematics, the MKD group showed higher knee internal rotation, knee abduction, and ankle abduction than the controls.							NA
[63]	1—sling open chain knee extension (SOCKE) exercise 2—sling closed chain knee extension (SCCKE) exercise	Muscle activation patterns (MVC), maximal voluntary contraction	Healthy subject	Students	Female: 7	NA	Female: 21.3 ± 0.6	Female: 23.94	Maximal voluntary contraction MVC							NA
									SOCKE exercise				SCCKE exercise			
									VMO (%)	VL (%)			VMO (%)	VL (%)		
									86.30 ± 14.61	86.20 ± 7.25			85.67 ± 6.27	77.85 ± 11.81		
[64]	“1. Back Squat (Double Legged) stable load on a stable surface, stable load on an unstable surface, unstable load on a stable surface”.	“Kinematics of the Ankle, Knee, Hip, Trunk, and the Bar/Attitude. Muscle activation patterns (EMG)”.	Healthy Subject	University population	Male: 10	NA	Overall: 21 ± 3	Overall: 24.1	During the squat movement, the Gluteus Medius muscle showed greater activity in the squat movement on the stable bar, squat on the tube, and squat in imbalance. The vastus medialis muscle, in turn, showed greater activity in the squat in imbalance, followed by the tube and bar. Represented in Figures 3 and 4 of the article [64]							NA

Abbreviation: Ref.: Reference; Ctr: Control; BMI: Body Mass Index; SR: Systematic Review; Mpa: (Mega Pascal); gender: gender distribution N: Newton; NA: Not applicable; MDK: medial knee displacement; CT: computed tomography.

**Table A2:** Studies quality assessment by NHLBI tool.

Ref.	[22]	[23]	[24]	[25]	[26]	[33]	[34]	[35]	[36]	[37]	[38]	[39]	[40]	[41]	[42]	[43]	[44]	[45]	[46]	[47]	[48]	[49]	[50]	[51]	[52]	[53]	[54]	[55]	[56]	[57]	[58]	[59]	[60]	[61]	[62]	[63]	[64]
**(a)**	X	X	X	X	X	X	X	X	X	X	X	X	X		X	X		X	X	X		X	X		X	X				X	X		X	X		X	X
**(b)**																					X			X											X		
**(c)**																													X								
**(d)**																																X					
**(e)**														X			X										X	X									
**Question**																																					
1	Y	Y	Y	Y	Y	Y	Y	Y	Y	Y	Y	Y	Y	Y	Y	Y	Y	Y	Y	Y	Y	Y	Y	Y	Y	Y	Y	Y	Y	Y	Y	Y	Y	Y	Y	Y	Y
2	Y	Y	Y	Y	Y	Y	Y	Y	Y	Y	Y	Y	Y	NR	Y	Y	Y	Y	Y	Y	Y	Y	Y	Y	Y	Y	Y	Y	Y	Y	Y	Y	Y	Y	Y	Y	Y
3	Y	Y	Y	Y	Y	Y	Y	Y	Y	Y	Y	Y	Y	NA	Y	Y	Y	Y	Y	Y	Y	Y	Y	N	Y	Y	Y	Y	Y	Y	Y	NA	Y	Y	Y	Y	Y
4	Y	Y	Y	Y	Y	Y	Y	Y	Y	Y	Y	Y	Y	NR	Y	Y	NR	Y	Y	Y	Y	Y	Y	Y	Y	Y	Y	NR	Y	Y	Y	NA	Y	Y	Y	Y	Y
5	NR	NR	NR	NR	NR	NR	NR	NR	NR	NR	Y	NR	NR	NR	NR	NR	NR	NR	NR	NR	Y	NR	NR	Y	NR	NR	Y	NR	N	NR	NR	Y	NR	NR	Y	NR	NR
6	Y	Y	Y	Y	Y	Y	Y	Y	Y	Y	Y	Y	Y	Y	Y	Y	Y	Y	Y	Y	Y	Y	Y	Y	Y	Y	Y	Y	Y	Y	Y	Y	Y	Y	Y	Y	Y
7	NA	NA	NA	NA	NA	NA	NA	NA	NA	NA	NA	NA	NA	Y	NA	NA	Y	NA	NA	NA	Y	NA	NA	Y	NA	NA	NA	Y	Y	NA	NA	Y	NA	NA	Y	NA	NA
8	Y	Y	Y	Y	Y	Y	Y	Y	Y	Y	Y	Y	Y	Y	Y	Y	Y	Y	Y	Y	Y	Y	Y	Y	Y	Y	Y	Y	NR	Y	Y	NA	Y	Y	Y	Y	Y
9	Y	Y	Y	Y	Y	Y	Y	Y	Y	Y	Y	Y	Y	Y	Y	Y	Y	Y	Y	Y	Y	Y	Y	Y	Y	Y	Y	Y	Y	Y	Y	Y	Y	Y	Y	Y	Y
10	NA	NA	NA	NA	Y	NA	NA	NA	NA	NA	NA	NA	NA	Y	NA	NA	Y	NA	NA	NA	Y	NA	NA	Y	NA	NA	NA	Y	Y	NA	NA	-	NA	NA	Y	NA	NA
11	Y	Y	Y	Y	Y	Y	Y	Y	Y	Y	Y	Y	Y	Y	Y	Y	Y	Y	Y	Y	NR	Y	Y	NR	Y	Y	Y	Y	Y	Y	Y	-	Y	Y	NR	Y	Y
12	NA	NA	NR	NA	NA	NR	NA	NA	NA	NA	NA	NA	NA	NR	NA	NA	NR	NA	NR	NA	NR	NR	NA	NR	NR	NA	Y	NR	NR	NA	NA	-	NR	NA	NR	NR	NA
13	Y	Y	Y	Y	Y	Y	Y	Y	Y	Y	Y	Y	Y	Y	Y	Y	Y	Y	Y	Y	-	Y	Y	-	Y	Y	Y	Y	-	Y	Y	-	Y	Y	-	Y	Y
14	Y	Y	Y	Y	Y	Y	Y	Y	Y	Y	Y	Y	Y	Y	Y	Y	Y	Y	Y	Y	-	Y	Y	-	Y	Y	Y	Y	-	Y	Y	-	Y	Y	-	Y	Y
**TOTAL**	10	10	10	10	11	10	10	10	10	10	11	10	10	9	10	10	11	10	10	10	10	10	10	9	10	10	12	11	9	10	10	6	10	10	10	10	10
**Result**	G	G	G	G	G	G	G	G	G	G	G	G	G	F	G	G	G	G	G	G	G	G	G	G	G	G	G	G	G	G	G	F	G	G	G	G	G

Legend: X ≥ 70% = GOOD, 70% > X ≥ 50% = FAIR, X < 50% = Poor; (a) Observational Cohort and Cross-Sectional Studies (14 question); (b) Case-Control Studies (12 question); (c) Before-After (Pre-Post) Studies With No Control Group (12 question); (d) Case Series Studies (9 question); (e) Controlled Intervention Studies (14 question).
